# The process of hip fracture management before and during the COVID-19 pandemic in Iran

**DOI:** 10.1186/s12877-024-04839-z

**Published:** 2024-04-23

**Authors:** Fatemeh Yalamchi, Kazem Khalagi, Noushin Fahimfar, Pouria Tabrizian, Mahnaz Sanjari, Mohammad Javad Mansourzadeh, Afshin Ostovar, Mohsen Asadi-Lari

**Affiliations:** 1 Department of Epidemiology, School of Public Health, Iran University of Medical Sciences, Tehran, Iran; 2 Osteoporosis Research Center, Endocrinology and Metabolism Clinical Sciences Institute, Tehran University of Medical Sciences, Tehran, Iran; 3 Obesity and Eating Habits Research Center, Endocrinology and Metabolism Clinical Sciences Institute, Tehran University of Medical Sciences, Tehran, Iran; 4 Department of Epidemiology and Biostatistics, School of Public Health, Tehran University of Medical Sciences, Tehran, Iran; 5 Bone and Joint Reconstruction Research Center, Shafa Orthopedic Hospital, Iran University of Medical Sciences, Tehran, Iran; 6 Endocrinology and Metabolism Research Center, Endocrinology and Metabolism Clinical Sciences Institute, Tehran University of Medical Sciences, Tehran, Iran

**Keywords:** Bone, Osteoporosis, Hip fracture, COVID-19 pandemic, Iran

## Abstract

**Background:**

The COVID-19 pandemic affected the control of many chronic conditions, including hip fractures, worldwide. This study was to examine the impact of the COVID-19 pandemic on the management of hip fractures in a referral orthopedic hospital in Iran. By understanding how the pandemic has influenced the care of hip fracture patients, we can gain valuable insights into the challenges, adaptations, and potential improvements in orthopedic healthcare during such public health crises.

**Methods:**

Data was collected on hip fracture patients aged 50 and above who were admitted to the hospital before and during the pandemic. The number of admissions and operations, length of hospital stay, and time from admission to surgery were recorded from the hospital information system (HIS) and compared between the two periods.

**Results:**

The median number of admitted hip fracture patients per month increased slightly during the pandemic (11%), although this increase was not statistically significant (*p* = 0.124). After adjusting for potential confounders, the mean length of hospital stay was significantly lower during the pandemic period, indicating that patients were discharged sooner (*p* = 0.019) and the time from admission to surgery was shorter during the pandemic (*p* = 0.004). Although the increase in the number of hip fracture surgeries per month during the pandemic was not statistically significant (*P* = 0.132), a higher percentage of patients underwent surgery during the pandemic compared to before (84.8% VS. 79.4%).

**Conclusion:**

The study suggests that the COVID-19 pandemic did not have a negative impact on hip fracture management in the investigated orthopedic hospital in Iran. further research is needed to explore the effects of the pandemic on other aspects of healthcare services, particularly in general hospitals.

## Introduction

The “COVID-19 pandemic” declared by the World Health Organization on March 11, 2020, refers to the global outbreak of an infectious disease caused by the novel β-coronavirus which was first identified in December 2019 in Wuhan City, the capital of Hubei province, China [1–4]. By July 25, 2023, more than 690 million people in the world were diagnosed as confirmed cases, and the number of mortality due to this disease exceeded six million nine hundred thousand. Also, the number of confirmed cases in Iran was more than 7 million people and 146,304 deaths were registered in the same period [5]. During the pandemic, the management of chronic conditions and the provision of medical services were partially disrupted across the world [6, 7], for example, the delivery of vital health services for non-communicable diseases (NCDs) in the WHO South-East Asia Region, such as cancer and cardiovascular diseases, was impacted [8]. In the same situation, the control of osteoporotic fractures in older adults was no exception [9]. Osteoporosis is a worrying public health challenge worldwide [10], and its most important symptom is fracture. The most common sites of osteoporotic fractures are the spine, hip, forearm and proximal humerus [11], among which hip fracture leads to the greatest morbidity and mortality [10]. Unfortunately, these fractures are increasing in the world and it is predicted that by the year 2050, the incidence of hip fracture worldwide will increase by 310% in men and 240% in women [12], which incurs the highest direct costs for health services among osteoporotic fractures; considering that their incidence increases exponentially with age [10]. Since hip fractures in Iran also lead to significant clinical and economic burden [13], we were seeking to answer the question that how the COVID-19 pandemic had affected the management of hip fractures of patients over 50 years old in an orthopedic referral hospital in Iran. When designing the study, we expected that the pandemic would have a negative impact on the overall performance of the healthcare system in dealing with hip fracture cases.

## Methods

### Study design

A descriptive-analytical cross-sectional study was conducted to compare the hospitalization services and surgical procedures of all patients aged 50 years and older diagnosed with hip fractures (based on the diagnostic codes of the International Statistical Classification of Diseases and Related Health Problems, 10th edition [ICD-10], including S72.00, S72.10 and S72.20) admitted to a Shafa Orthopedic Hospital (a referral orthopedic teaching hospital in Tehran) before and during the COVID-19 pandemic, that was from February 20, 2019, to February 19, 2020 (before the COVID-19 pandemic) and from February 20, 2020, to November 21, 2021 (during the COVID-19 pandemic). During this period, Iran experienced five peaks of the pandemic, the time intervals of the peaks were determined by matching the cases confirmed in the report of the World Health Organization and the cases of hospitalization in the report of the Ministry of Health of Iran. We divided the causes of fracture into 4 categories based on the information available in the patient’s records including same-level falls, upper-level falls, trauma, and no history of trauma. In addition, we divided hip fractures into the neck of femur, intertrochanteric, subtrochanteric, both neck of femur and intertrochanteric, both intertrochanteric and subtrochanteric based on the location of the fracture. Patients with pathological fractures and complications due to previous fractures were excluded from the study. It is worth mentioning we have identified all new hospitalizations that received the ICD10 code associated with hip fractures in the hospital information system. However, after reviewing the patient files, we discovered that some of these hospitalizations were a result of complications arising from previous fractures; therefore, we excluded them from the study.

### Data source

The treatment process of hip fractures data such as the number of hospitalized cases, length of stay, the interval between admission and surgery, frequency of hip surgeries, frequency of hip fracture surgical site infection, and frequency of re-operation were collected from patient record information using the hospital information system in the selected hospital in Tehran, the capital of Iran. The hospital records of all patients who were admitted to this hospital on the desired dates and underwent surgery were monitored for two months after their surgery, and all cases of infection and re-surgery during this period were extracted. Patients who underwent hip surgery again after the initial surgery for any reason were considered re-surgery cases.

### Statistics

Data obtained in the study were analyzed with IBM SPSS statistics software (IBM SPSS Statistics v26). The variables were checked for normal distribution with normality plots and normality tests. Continuous quantitative variables were described with a mean (Standard deviation: SD) and median (Interquartile range: IQR), in variables with outlier data, for a better description. Qualitative variables were reported as percentages. Normally distributed variables were compared with Student’s t-test concerning the group number. Non-normally distributed variables were evaluated with Mann–Whitney U test. The Chi-square test or the Fisher Exact test was applied to the categorical variables. A value of *P* < 0.05 was considered statistically significant. In this study, potential confounding factors, including age, sex, residence out of Tehran province and the comorbidity of at least one chronic disease such as diabetes, heart disease and stroke, chronic kidney disease, chronic obstructive pulmonary disease (COPD) and cancer were controlled with regression models.

In linear regression models, the logarithm of outcome variables was used to approach normal distribution for the residuals.

## Results

In general, 672 patients 50 years and older were hospitalized with a diagnosis of hip fracture in the study periods; 38 of them were with pathological causes or complications due to previous fractures, who were excluded from the study, and finally, 634 patients analyzed (Table 1). The mean age of the patients in the pre-pandemic period were 72.30 ± 10.9 years and 73 ± 10.9 years (P-value = 0.4) in the pandemic period. In the pre-pandemic period, 114 (57.3%) patients were women, while during the pandemic period, 235 (54%) women were admitted (P-value = 0.4), no significant (Table 1). The number of patients with hip fracture admitted to the hospital increased by 11% per month during the pandemic period (Table 2), however, it was not statistically significant. In total, 158 cases (79.4%) of patients were operated before the pandemic compared to 369 cases (84.8%) during the pandemic (*P* = 0.132) (Table 1). The number of hip fracture surgeries per month was not significantly different between the two periods (Table 2). Meanwhile, we examined the number of hip fracture surgical site infection up to two months after the surgery and the number of cases of hip fracture re-surgery between the two periods of our study, which were not statistically different (Table 2).

Table 3 shows that the mean of hospital stay was 5.65 ± 3.55 days in the pre-pandemic period which was significantly higher than in the pandemic period 5.01 ± 3.36 days, adjusted for potential confounders such as age, sex, residence out of Tehran province, comorbidity of diabetes, cardiovascular diseases, history of stroke, chronic kidney disease, chronic obstructive pulmonary disease, and cancer (*P* = 0.019). Likewise, the mean ± SD time from admission to surgery was 3.63 ± 2.5 days in the pre-pandemic and 3.92 ± 9.7 days in the pandemic period, but it was significantly longer than the pandemic period after adjusting for potential confounders (*p* = 0.004) (Table 4).

**Table 1:** Characteristics of the hip fracture patients before and during the COVID-19 pandemic

Variable	Pre-pandemic period (2019.02.20 to 2020.02.19) *N* = 199			Pandemic period (2020.02.20 to 2021.11.21) 435	P-value
Age [mean (SD)]	72.3 (10.92)			73 (10.9)	0.45
Sex Female (%)	114 (57.3%)			235 (54%)	0.44
**Fracture site (%)**					0.91
Neck of femur	95 (47.7%)			161 (37%)	
Intertrochanteric	87 (43.7%)			225 (51.7%)	
Subtrochanteric	6 (3%)			22 (5.1%)	
Neck of femur & Intertrochanteric	6 (3%)			10 (2.3%)	
Intertrochanteric & Subtrochanteric	5 (2.5%)			17 (3.9%)	
**Cause of fracture (%)**					0.26
Same level fall	152 (76.4%)			353 (81.1%)	
Upper-level fall	27 (13.6%)			57 (13.1%)	
Trauma	12 (6%)			16 (3.7%)	
No history of trauma	8 (4%)			9 (2.1%)	
Place of residence (%) Tehran	181 (91%)			399 (91.7%)	0.68
COVID-19 based on PCR diagnostic test (%)	–			10 (2.3%)	–
Comorbidity^c^ (%)	94 (47.2%)			226 (52%)	0.27
Patients who underwent surgery (%)		158 (79.4%)	369 (84.8%)		0.13

^c^Comorbidity of diabetes, cardiovascular diseases, history of stroke, chronic kidney disease, COPD, cancer

**Table 2:** Process of hip fracture management before and during the COVID-19 pandemic

Variable	Pre-pandemic period (2019.02.20 to 2020.02.19) *N* = 199	pandemic period (2020.02.20 to 2021.11.21) 435	P-value	
Number of hip fracture admitted per month [median (IQR)]	18 (5)	20 (10)	0.124^m^	
Duration of staying in the hospital-Day [mean (SD)]	5.65 (3.55)	5.01 (3.36)	**0.032** ^i^	
Interval between admission to surgery-Day [median (IQR)]	3 (3)	2 (3)	***< 0.001*** ^m^	
Number of hip fracture surgeries per month [median (IQR)]	14 (5)	16 (8)	0.68^m^	
Number (%) of Surgical site infection of hip fractures ^u^	5 (3.2%)	4 (1.1%)	0.14^f^	
Number (%) of re-surgery of hip fractures ^u^	7 (4.4%)	11 (3%)	0.4^c^	

IQR: interquartile range. SD: standard deviation

^i^Independent Sample Test

^m^Mann-Whiney Test

^c^Chi-Square Test

^f^Fisher’s Exact Test

^u^Up to two months after the first surgery

**Table 3:** Effect of the COVID-19 pandemic on the duration of staying in hospital after adjusting for the potential confounders

Variable	Log-linear regression coefficient(b) (95% confidence interval)	P-value
COVID-19 pandemic (Pandemic period)	−0.130(−0.239, −0.022)	***0.02*** ^*^
Age (Year)	0.000(-0.004, 0.005)	0.88
Sex (Male)	−0.035(−0.138, 0.068)	0.50
Residence (Out of Tehran province)	0.012(−0.076, 0.100)	0.79
Comorbidity^c^ (Yes)	−0.005(−0.107, 0.097)	0.92

^c^Comorbidity of diabetes, cardiovascular diseases, history of stroke, chronic kidney disease, COPD, cancer

^*^Statistically significant < 0.05

**Table 4:** Effect of the COVID-19 pandemic on the interval between admission to surgery after adjusting for the potential confounders

Variable	Log-linear regression coefficient(b) (95% confidence interval)	P-value
COVID-19 pandemic (Pandemic period)	−0.202(−0.340, −0.065)	***0.004*** ^*^
Age (Year)	0.003(−0.003, 0.009)	0.34
Sex (Male)	−0.058(−0.188, 0.072)	0.38
Residence (Out of Tehran province)	−0.011(−0.114, 0.091)	0.83
Comorbidity ^c^ (Yes)	0.062(−0.067, 0.191)	0.35

^c^Comorbidity of diabetes, cardiovascular diseases, history of stroke, chronic kidney disease, COPD, cancer

^*^Statistically significant < 0.05

## Discussion

The COVID-19 pandemic affected all aspects of the population’s life, nonetheless, its effects on chronic conditions and even some medical emergencies might have more implications on people’s lives. The study sought to examine the impact of the COVID-19 pandemic as a public health emergency on hip fracture management in individuals aged 50 years and above, aiming to gather insights into the potential effects of future emergencies.

The number of admitted hip fracture patients per month increased during the pandemic compared to the pre-pandemic period, which was not statistically significant. Several reasons can support such an increment: probably, during the pandemic, orthopedic patients were directed to the tertiary referral hospitals focused on providing specialized care for orthopedic patients to prevent further transmission of infection or patients and their families used to go to trauma centers due to the fear of transmission of infection in general hospitals and for this reason, the number of admissions increased in this period. In fact, during the COVID-19 pandemic, general hospitals had to allocate a significant portion of their inpatient capacity to treat COVID-19 patients. Consequently, it was predictable that certain orthopedic patients would be referred to the referral hospital for their treatment needs. It is worth mentioning that this hospital is a specialized referral center that accepts patients from across the country and admission of patients in this hospital is common, both through direct visits and referrals from healthcare facilities nationwide. The increasing number of hip fractures in older adults during the pandemic was not surprising, because the majority of hip fractures in older adults are due to falls [14, 15] and more than half of the falls happen in homes or nearby [16], therefore, social restrictions and prohibition of movement outside the home, as well as lockdowns during the pandemic, have not had a reducing effect on the occurrence of hip fractures among older adults. On the other hand, we found that less communication with seniors while social distancing during the pandemic might have caused the loneliness of the older adults [17]. In fact, the act of staying at home alone and performing tasks independently can increase the risk of falls and subsequently, the likelihood of experiencing a hip fracture among older adults. A couple of studies in the literature have shown similar results in agreement with the increased incidence of hip fractures during the COVID-19 pandemic [18, 19]. Even in some studies that have reported a decrease in other orthopedic and trauma patients (such as all bone fractures, dislocations, etc.,) during the pandemic, an increase in osteoporosis-related fractures in older adults has been observed during the COVID-19 era [20, 21]. In the meantime, some studies reported a decrease in hospitalizations or even a decrease in the incidence of hip fractures during the pandemic [22, 23]. Alternatively, stability in the number of hip fractures in older adults during the pandemic was observed, too [24, 25]. A cohort study, limited to the first COVID-19 lockdown in 2020, was conducted in French national hospitals and showed that the total number of hip fractures decreased during this period compared to the same period in 2019 [22], this contradictory finding with ours could be due to the longer duration of our study. Another study to compare the incidence of hip fractures before and during the COVID-19 pandemic in Brazil indicated a decrease in the incidence of hip fractures during the pandemic [23], which was because all hip fractures services provided by the Brazilian public health care system were included in that study, while we only studied hip fracture patients in one of the major referral orthopedic centers.

Reduction in duration of hospitalization and duration of hospitalization until surgery were other findings raised in our research. Similar to our study, in Northern Ireland, the USA and the East Midlands region of England a shorter length of stay during the pandemic was reported compared to the pre-pandemic period [26–28]. Also, the shorter time to surgery during the pandemic was a finding that other studies have supported [29, 30]. However, several studies have also pointed to the extended length of the pre-operative time interval during the pandemic [31, 32]. In a study conducted in South Korea, no significant difference was found in these two variables between the pandemic and pre-pandemic periods [33].

Hip fracture is life-threatening and is considered as an orthopedic emergency and according to the guidelines the patient must be hospitalized to receive different treatments. During the COVID-19 pandemic, attention was focused on reducing the number of hospitalized patients and shortening their hospitalization time and hospitalized patients were discharged from the hospital at the earliest safe time [34]. Thus, the reduction of these two variables during the pandemic in our study could be an indication of the correct implementation of protocols related to the pandemic in the hospital. On the other hand, the shortening of the patient’s hospitalization time can itself be caused by the reduction of the interval between diagnosis and surgery [35]. In addition, it is important not to overlook the possibility of reducing other fractures, such as those caused by accidents during the pandemic, due to social restrictions, by reducing the overall burden of patient visits in the hospital, staff and doctors could focus their attention on providing faster and more efficient services to the patients who were admitted. This could have led to better care for those patients.

Our study showed that the frequencies of surgeries, surgical site infection, and re-surgeries did not show a significant variation in the two time periods before and during the pandemic, which was similar to other studies [36, 37]. In the United Kingdom, there was no significant difference in the rate of postoperative complications, including infection [36]; however, another study reported fewer postoperative infections during the pandemic [38]. In another study in Camden, New Jersey, the research team also did not find a difference in re-surgery; however, the age of the people included in their study was different from our study [37]. The findings of our study about these three variables showed that despite the increase in the number of patients and the distraction of public attention to the pandemic emergency, it had no adverse effect on the care of patients with hip fracture in this referral hospital; and hospital services for hip fracture patients over 50 years old during the COVID-19 pandemic had not deviated from its original path.

### Limitations

We acknowledge the limitations of our study, which include cross-sectional design, single-center study site and also the absence of variables that may have influenced the study results (e.g., living alone or lacking family support, income and economic status and type of medical insurance). Moreover, our study was limited to the fact that some patients were referred to other hospitals due to pulmonary or cardiovascular conditions after being hospitalized in this referral fracture hospital.

## Conclusion

The COVID-19 pandemic did not negatively affect hip fracture management in the referral hospital that we investigated in Iran. However, further research is needed to investigate other aspects of services, especially in general hospitals. Our study showed that non-communicable diseases such as hip fractures in older adults remain or may even increase in the pandemic situation, so some measures are necessary to preserve such essential health services in future crises. Considering that the issue of emerging and re-emerging infectious diseases is a constant problem of public health, health decision-makers must be careful not to neglect the management of non-communicable diseases that have long-term consequences when managing challenges such as the COVID-19 pandemic.

## Data Availability

The data that support the findings of this study are available from Shafa hospital information system but restrictions apply to the availability of these data, which were used under license for the current study, and so are not publicly available. Data are however available from the authors upon reasonable request and with permission of Shafa hospital information system.

## Abbreviations

HIS: Hospital information system
COPD: chronic obstructive pulmonary disease
